# Ruptured Hemorrhagic Ovarian Cyst Presenting as an Incarcerated Inguinal Hernia in an Adult Female: A Rare Clinical Scenario of a Common Surgical Emergency

**DOI:** 10.1155/2013/925694

**Published:** 2013-04-18

**Authors:** Priyadarshan Anand Jategaonkar, Sudeep Pradeep Yadav

**Affiliations:** ^1^Department of Surgery, Mahatma Gandhi Institute of Medical Sciences, Sevagram, Wardha, Maharashtra 442102, India; ^2^Department of Surgery, Jagjivanram Western Railway Hospital, Mumbai Central, Mumbai, Maharashtra 400008, India

## Abstract

Embryoanatomical peculiarities are responsible for low occurrence of inguinal hernias in females. Amongst them, ovarian hernias are rarer. They are commonly noticed in children. An attending surgeon commonly faces diagnostic and operative dilemmas in managing these overtly “simple-looking” clinical scenarios. Although ovarian cysts are one of the common contents of the sac, we report a case of adult incarcerated ovarian hernia who presented with a ruptured hemorrhagic ovarian cyst. This differential should be kept in mind while treating an adult female with painful inguinal swelling. As far our knowledge goes, such case with ruptured ovarian cyst presenting as an incarcerated hernia in an emergency scenario has not been reported as yet.

## 1. Introduction

Groin hernia is one of the most common disorders tackled by any general surgeon. Inguinal hernia is the commonest subtype in females. Though almost every pelvic organ has been described as the content of the inguinal sac, ovarian hernias remain one of the rarer subtypes (incidence = 2.9%) usually seen in pediatric population [1, 2]. 

Although ultrasonography is helpful in preoperative diagnosis, it still may be a “surprise finding” on the operation table in sizeable number of cases. We describe a case presenting as incarcerated inguinal hernia with ruptured hemorrhagic ovarian cyst as the content—a rare clinical scenario. 

## 2. Case

33-year-old female patient came to our office with 2-day history of painful left inguinal swelling. A detailed history taking revealed that it was a reducible swelling present since birth. She denied any history of irreducibility in the past. As it was otherwise asymptomatic, she never bothered to consult any physician. She never had any trauma at that site. Local examination revealed a globular, tender 4 × 4 cm irreducible swelling without an expansible impulse on coughing. It was situated above the inguinal skin crease. Ultrasound imaging revealed a 4 × 3.4 cm adnexal mass in the inguinal canal; it had a mixed echotexture with suspicious absence of blood flow on Doppler assessment. There was a collection of fluid around the mass. 

**Figure 1:**
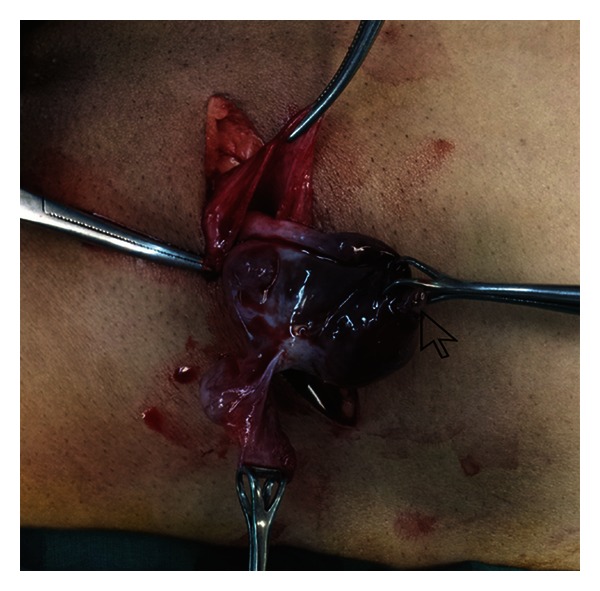
Ruptured hemorrhagic cyst as the content of inguinal hernia. Note the pouting mucosa at the site of rupture (arrow) and the spilled hemorrhagic fluid in the sac.

With the clinical diagnosis of incarcerated hernia, she was taken up for emergency surgery. On exploration of the inguinal region, we found a tense, irreducible inguinal hernia with a bluish discoloration of the sac. On opening, the sac was found to be full of hemorrhagic fluid secondary to a ruptured hemorrhagic ovarian cyst. The site of rent had the mucosa pouting out (Figure 1). The ipsilateral Fallopian tube with a small cyst was amongst the other contents. Ovarian cystectomy with herniorrhaphy was performed. Mesh repair was deferred in this case as there was a possibility of its infection due to the collected hemorrhagic fluid from the cyst. Postoperative recovery of the patient was good. Histopathology confirmed that it was a hemorrhagic ovarian cyst.

## 3. Discussion

Ovarian hernias are usually present in pediatric patients; they rarely occur in adulthood especially in premenopausal females [2]. Weakness of supporting ligaments of ovary coupled with increased intra-abdominal pressure forms the basis of its occurrence [3]. They are at risk of torsion and gangrene if left untreated. Hence, tackling them at the earliest becomes mandatory even when they are asymptomatic. Although ultrasonography can clinch the preoperative diagnosis [4], the exact finding may remain uncertain until the time of operation. Further, we feel that the imaging facility may not always be handy especially in an emergency situation at an odd working hour in a rural hospital setting. 

Moreover, as seen in our patient, the hemorrhagic cyst can rupture and mimic strangulation. Thus, the general surgeon should be aware of such complication to circumvent any “on-table” surprises and should be ready with the management strategies. Unless gangrenous, an organ-conserving surgery is always a better option. And, to avoid mesh infection, we feel that it is better to perform pure tissue repair in such emergent situations. Although laparoscopic management of ovarian hernia is described in literature [5], we feel that it may be avoided in such an emergency situation. 

## 4. Conclusion

Though rare, ruptured hemorrhagic cyst should be kept in mind while treating an incarcerated ovarian hernia in an adult premenopausal female. Emergent surgical exploration with ovarian cystectomy is the preferred option. While the use of mesh should be avoided in this tricky situation, a good pure tissue repair is the key to successful outcome.
